# High Emergence of ESBL-Producing *E. coli* Cystitis: Time to Get Smarter in Cyprus

**DOI:** 10.3389/fmicb.2015.01446

**Published:** 2016-01-13

**Authors:** Leon Cantas, Kaya Suer, Emrah Guler, Turgut Imir

**Affiliations:** ^1^MicroLabHammerfest, Norway; ^2^Department of Medical Microbiology, Faculty of Medicine, Near East UniversityNicosia, Cyprus; ^3^Department of Infectious Diseases and Clinical Microbiology, Faculty of Medicine, Near East UniversityNicosia, Cyprus

**Keywords:** ESBL, *E. coli*, antibiotic, resistance, UTI, Cyprus

## Abstract

**Background:** Widespread prevalence of extended-spectrum βeta-lactamase producing *Escherichia* coli (ESBL-producing *E. coli*) limits the infection therapeutic options and is a growing global health problem. In this study our aim was to investigate the antimicrobial resistance profile of the *E. coli* in hospitalized and out-patients in Cyprus.

**Results:** During the period 2010–2014, 389 strains of *E. coli* were isolated from urine samples of hospitalized and out-patients in Cyprus. ESBL-producing *E. coli*, was observed in 53% of hospitalized and 44% in out-patients, latest one being in 2014. All ESBL-producing *E. coli* remained susceptible to amikacin, carbapenems except ertapenem (in-patients = 6%, out-patients = 11%).

**Conclusion:** High emerging ESBL-producing *E. coli* from urine samples in hospitalized and out-patients is an extremely worrisome sign of development of untreatable infections in the near future on the island. We therefore emphasize the immediate need for establishment of optimal therapy guidelines based on the country specific surveillance programs. The need for new treatment strategies, urgent prescription habit changes and ban of over-the-counter sale of antimicrobials at each segment of healthcare services is also discussed in this research.

## Introduction

Urinary tract infections (UTIs) called cystitis is one of the most common bacterial infection in humans and *Escherichia coli* (*E. coli*) causes the vast majority of UTIs worldwide (Picozzi et al., 2014). Furthermore, UTI-causing bacteria is becoming more resistant to available antimicrobials with the increased incidence of Extended-Spectrum Beta-Lactamase (ESBL) since its first detection in1980s in Germany, shortly after the use of the oxyimino β-lactam drugs (Rice et al., 1990; Livermore and Hawkey, 2005). The ESBL strains are associated with resistance to amino and ureidopenicillins, oxyiminocephalosporins and monobactams, which are the most commonly used drugs in the treatment of various bacterial infections (Bradford, 2001; Kim et al., 2002; Kotra et al., 2002). ESBL genes are generally transmissible and they can be acquired between bacteria by horizontal gene transfer mechanism, mainly using conjugation. The most common genetic variant of ESBL is CTX-M (Paterson and Bonomo, 2005).

The ESBL-producing *E. coli* is isolated from cystitis both in hospitalized and out-patients and is increasingly posing significant therapeutic challenges (Hyle et al., 2005). It is causing greater use of other expensive antimicrobials (such as carbapenems), prolonged hospital stay, increasing morbidity, mortality and health care costs (Mehrgan and Rahbar, 2008).

Antimicrobial resistance and its spread is increasing due to misuse or overuse of antimicrobials, while the discovery of the potential novel antimicrobials has slowed drastically in last decade (Cantas et al., 2013). Most of the developed countries have recognized the importance of the acquired antimicrobial resistance surveillance programs to track the changes in the antimicrobial susceptibility of certain public health threatening pathogens to devise appropriate strategies for their control (NORM/NORM-VET, 2013; ECDC, 2014). In particular, the incidence of ESBL-producing organisms is difficult to resolve at the wider geographic scale level, mostly due to difficulty in detecting ESBLs and inconsistencies in reporting (Steward et al., 2000). Recently, an obvious increase in the prevalence of multi drug resistant (MDR) and ESBL-producing *E. coli* isolates from human sources has been observed throughout the globe (Gupta, 2007; Cantón et al., 2008; Fatemeh et al., 2012; Lu et al., 2012; Allocati et al., 2013; Picozzi et al., 2013). From a European aspect; the highest prevalence was in Bulgaria, Slovakia, and Italy (22–36%); whereas the lowest occurrence of the MDR *E. coli* were found to be in Sweden, Norway, and Finland (3–5%). Recently, the ESBL-producing *E. coli* prevalence was found to be about 15% in Eastern Europe including Turkey with the highest percentage resistance (25.2%; Balode et al., 2013). On the other hand; southern Cyprus had the highest third generation cephalosporin resistant *E. coli* occurrence (38.9%) in 2014; meanwhile it was found to be lowest in Scandinavian countries (i.e., Sweden with 3%; ECDC, 2014). The broad extent of the ESBL-producing Enterobacteriaceae distribution in the parts of Africa, Asia and the Indian subcontinent have lately begun to be understood with the increasing number reports. The occurrence of the ESBL-producing *E. coli* increased in different parts of China (13–35%; Hawkey, 2008) and Africa (35–65%; Ruth et al., 2011). Besides, incredibly high emerge of the ESBLs prevalence up to 80% in India is worrying (Nasa et al., 2012).

The frequent identification of ESBL-producing *E. coli* from urine samples prompted our interest to investigate the resistance profile of *E. coli* in hospitalized and out-patients with cystitis in Cyprus. To our knowledge, there are no known previous studies on these issues on the island. The interventions needed to meet the challenge are discussed in this study, as well.

## Materials and Methods

During the period 2010–2014, 389 strains of *E. coli* were isolated from urine samples of hospitalized and out-patients in Cyprus (**Table 1**). Urine samples (50 mL) were collected in universal container. The samples inoculated using an inoculating loop of 10 μL volume calibration on blood agar and EMB mediums that incubated overnight at 37°C in the Microbiology Laboratory of the Near East University Hospital (Nicosia, Cyprus). Samples were further examined with the BD Phoenix 100 Automated Microbiology System (Becton Dickson, USA) and Oxoid combination disk test methods. The inoculated Phoenix^TM^ panels were placed into the Phoenix^TM^ instrument for incubation and continuous reading. The following antimicrobial agents were used in the Phoenix^TM^ ESBL test: Ceftazidime, Ceftriaxone, and Ceftazidime. The ESBL result was determined based on all the responses within 5–11 h.

**Table 1:** The number of *Escherichia coli* isolates each year, cultured from urine samples of hospitalized and out-patients.

	Study population	
Year	In-patient	Out-patient
2010/2011	29	46
2012	36	55
2013	40	66
2014	45	72
Sum	150	239

Antimicrobial sensitivity records for each isolate yearly were coded in a Microsoft Excel 2013^®^ spreadsheet and the percentage (%) antimicrobial resistance displayed as a histogram (Cantas et al., 2011). Changes in resistance prevalence over time within in-patients and out-patients were assessed by chi-square tests.

## Results and Discussion

**FIGURE 1:**
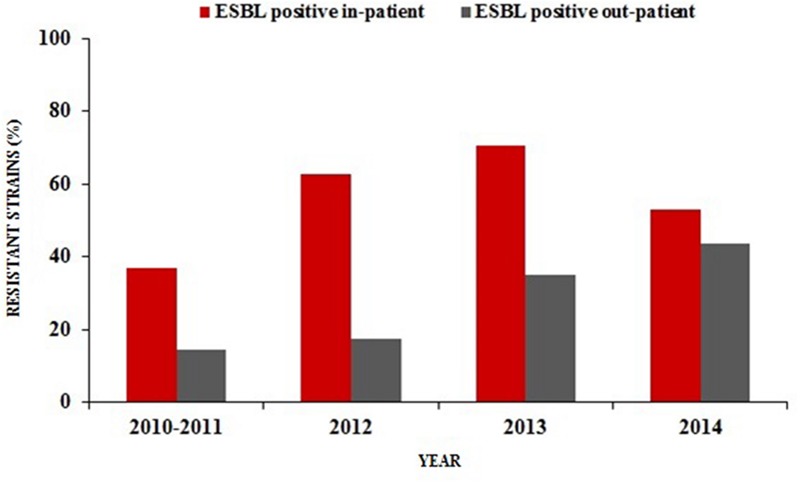
**Percentage distribution of ESBL-producing *Escherichia coli* in urine samples of hospitalized patients and out-patients (2010/2011–2014)**.

The prevalence of ESBL-producing *E. coli* was found to be relatively higher in hospitalized patients than out-patients during the last four years in urine culture isolates. The UTIs rate caused by ESBL-producing *E. coli* among hospitalized patients increased from 36% in 2010–2011 to 53% in 2014 with a significant rise of up to 71% in 2013 (*p* < 0.001). However, a gradual upward trend of ESBL-producing *E. coli* frequencies were also observed from 2010–2011 (14%) to 2014 (44%; *p* < 0.001) in UTIs of out-patients (**Figure 1**).

Frequent isolation of ESBL-producing of *E. coli* typically took place in hospital settings (Rodríguez-Baño et al., 2004; Livermore, 2007). Hospitalization, recurrent UTIs, catheter applications (biofilm formations) and previous antimicrobial treatment (especially with third-generation cephalosporins) or previous international travel were previously described as great risk factors for the acquisition of these organisms (Rodríguez-Baño et al., 2004; Woodford et al., 2004; Laupland et al., 2008; Topaloglu et al., 2010; Kang et al., 2012). Inevitably, these microbes today have begun to disseminate into the community worldwide (Colodner et al., 2004; Rodríguez-Baño et al., 2004). We have witnessed a recent 2.5-fold increase in the community-onset UTIs due to ESBL-producing *E. coli* in our region since the years 2010–2011 (**Figure 1**). These results are in line with recent reports (Coque et al., 2008; Topaloglu et al., 2010; Doi et al., 2013; Ansari et al., 2015) which highlight the rapid spread of these strains in the community. However, the proportion of ESBL-producing *E. coli* from out-patients with cystitis were found to be only 2.1% in Norway, a country with the lowest levels of antimicrobial consumption rates among the European countries (ESAC, 2009; NORM/NORM-VET, 2013).

The high resistance rate among out-patients in this study seems to be the result of widespread antimicrobial usage in Cyprus without prescription requirements especially in the northern part of the island. The actual defined daily doses (DDDs) of antimicrobials were not known *per se* during this study. However, earlier sale trends of systemic anti-infective agents in Cyprus revealed that there was one of the highest values in comparison with other European countries (Hadjimichael et al., 2006; ESAC, 2009). Another independent cross-sectional study showed that 97,6% of community pharmacists in the north of Cyprus engaged in inappropriate antimicrobial dispensing without medical prescriptions (Kaya Suer et al., 2015, unpublished survey). Furthermore, 60% of the physicians adhered to international antibiotic prescribing guidelines (Cantas, 2014, unpublished survey) that may not be implemented in parallel with nation specific epidemiological data in Cyprus. As resistance is becoming more widespread, prudent use of antimicrobials has to be supervised. Prescribers should prioritize diagnostics in order to make more targeted antimicrobial treatment decisions.

**FIGURE 2:**
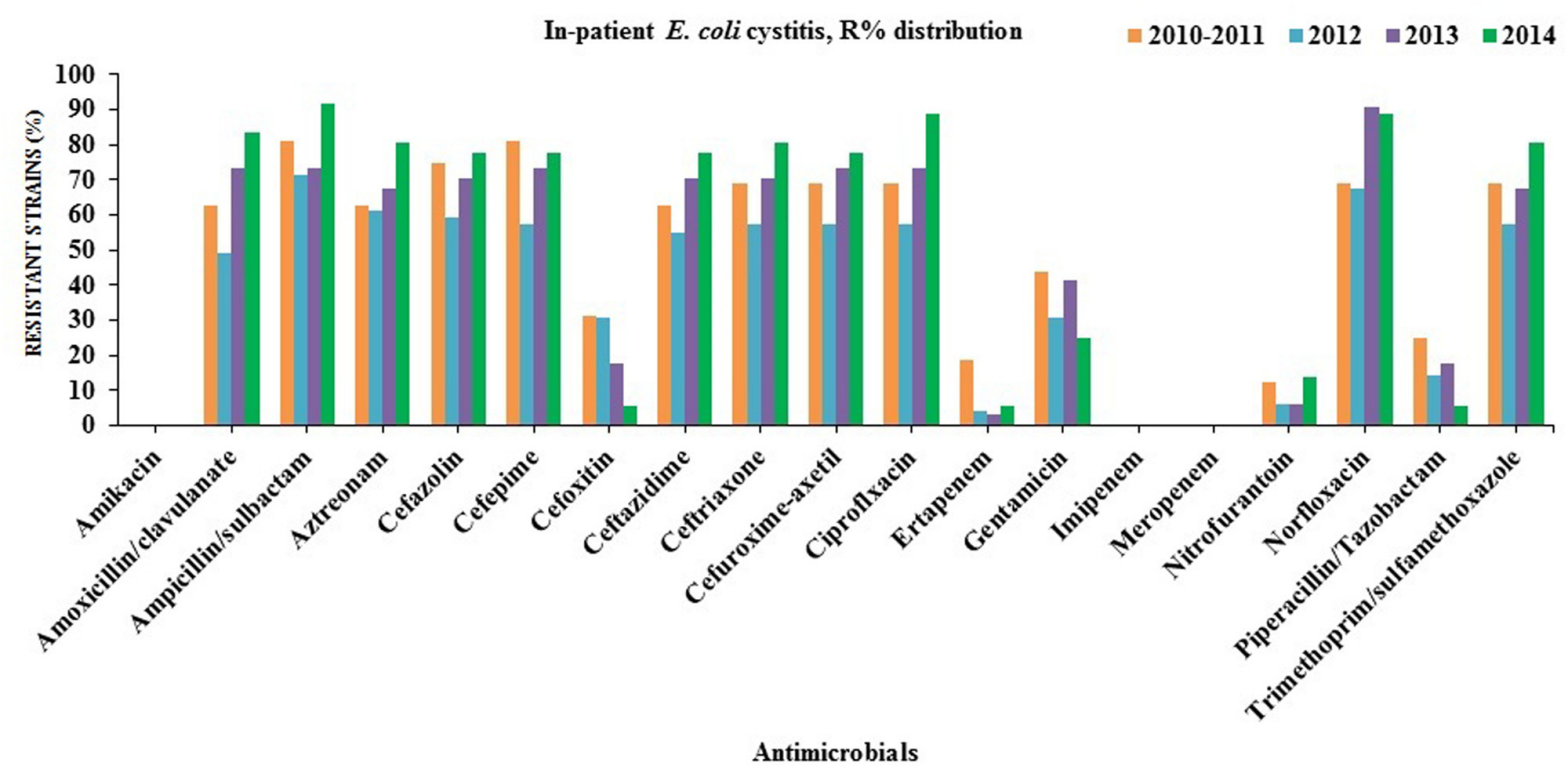
**Antibiotics susceptibility testing for all *E. coli* strains isolated from in-patients with cystitis (2010/2011–2014)**.

**FIGURE 3:**
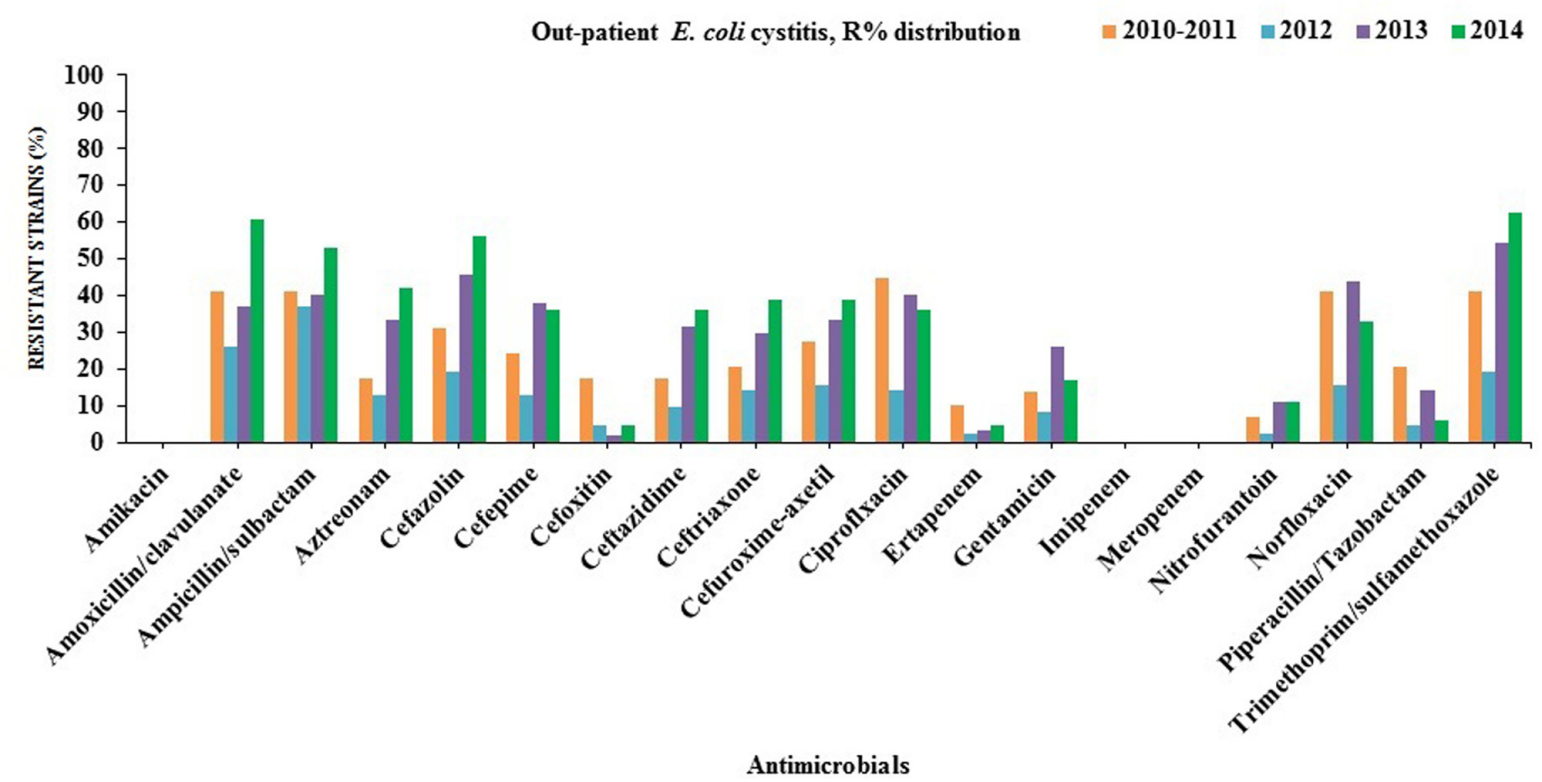
**Antibiotics susceptibility testing for all *E. coli* strains isolated from out-patients with cystitis(2010/2011–2014)**.

Many of the ESBL-producing *E. coli* isolates were found to be resistant to quinolones (ciprofloxacin and norfloxacin; in-patients = 78%, out-patients = 79%), gentamicin (in-patients = 45%, out-patients = 61%) and trimethoprim/sulfamethoxazole (in-patients = 60%, out-patients = 62%). On the contrary, all ESBL-producing *E. coli* remained susceptible to amikacin, carbapenems (imipenem and meropenem) except ertapenem (in-patients = 6%, out-patients = 11%). Partial resistance to nitrofurantoin (in-patients = 14%, out-patients = 11%) was also observed (**Figures 2** and **3**).

Non-resistance to meropenem and imipenem might be due to the limited usage of these antibiotics in northern Cyprus. Nitrofurantoin is one of the oldest urinary anti-infective drugs that have been widely used on the island. It has multiple action mechanisms on bacteria and demand several mutations in order to develop antimicrobial resistance that might explain its low prevalence in this region.

All other non-ESBL-producing *E. coli* were sensitive to imipenem, meropenem, amikacin, and mostly to nitrofurantoin (in-patient = 91%, out-patient = 93%). The highest resistance rates were against trimethoprim-sulfamethoxazole (in-patient = 69%, out-patient: 41%) in the years 2010–2011 to 2014.

Antimicrobial resistance to cephalosporins (>50%, except cefoxitin) in northern Cyprus was found to be significantly higher than in the Scandinavian countries (Sweden, Norway, and Finland; 3–5.1%) which have more restricted antimicrobial consumptions compared to other eastern European countries such as Bulgaria (22.9%) and Slovakia (31%) (Allocati et al., 2013). Cephalosporins have been frequently used for the empirical treatment of UTIs which shows a clear evidence of a strong relationship between prescribing habits and antimicrobial resistance (Lindbäck et al., 2010; Allocati et al., 2013).

Reduced susceptibility to both ciprofloxacin (53%) and gentamicin (26%) leaves clinicians only the choice of carbapenem in such serious cystitis treatment on the island. The fluoroquinolone resistance range was found to be significantly lower in Sweden (8%) and Norway (9%) (European Centre for Disease Prevention and Control [ECDPC], 2012), whereas it was found to be 42% in northern Cyprus. Furthermore, the prevalence of isolates resistant to aminoglycosides (amikacin and gentamicin) was found to be around 2% in this study whereas it was 4% in Sweden, 17% in Romania, Slovakia and Greece. However, the prevalence of multi-resistant (≥3 drugs) non-ESBL *E. coli* isolates were 24% in northern Cyprus, which recently were discovered to range from approximately 1% in Sweden to 10% in Romania and Slovakia.

The rise of ESBL-producing *E. coli* may lead to an increased consumption of carbapenems especially ertapenem due to the fact that it is administered only once daily, unlike the other carbapenems (Prakash et al., 2009). On the other, it facilitates the emergence and spread of carbapenemases. This is a great threat for public health and compels exploration of alternative therapeutic options. Herein, Cefoxitin has been recently suggested by (Raphaël et al., 2012; Guet-Revillet et al., 2014) as an alternative to carbapenems for the treatment of UTIs caused by ESBL-producing *E. coli*. Over 90% of the ESBL-producing *E. coli* isolates from hospitalized and out-patients were found to be sensitive to cefoxitin for the last 2 years (2013–2014) that might be recommended for the therapy of complicated cystitis rather than carbapenems as the first choice in northern Cyprus (**Figures 2** and **3**).

There is a limited number of practical solutions for the treatment of multi-resistant gram-negative bacteria. Non-antimicrobial prescription is recommended to suppress bacteriuria in the elderly without clinical signs of UTI (Raphaël et al., 2012; Guet-Revillet et al., 2014).

Extended courses of antimicrobials due to complicated cystitis should only be used in specific situations such as for men with a relapsing infection in prostate (Williams and Schaeffer, 2004).

The oral options available for the treatment of complicated UTIs caused by ESBL-producing *E. coli* with concurrent resistance to trimethoprim and quinolones are limited. In case of susceptibility, nitrofurantoin treatment can be recommended for lower UTIs but resistance may develop upon treatment (Pallett and Hand, 2010; Cantas, 2014, unpublished survey). Rather, some immune-modulating cranberry, pre-probiotic products are recommended to reduce the frequency of recurrent UTIs (Williams and Schaeffer, 2004; Wagenlehner et al., 2005; McMurdo et al., 2009).

It is known that a combination of antimicrobials particularly cephalosporin with clavulanic acid has been used to treat UTIs caused by CTX-M ESBL-producing *E. coli* in clinical practice (Livermore et al., 2008), which are unlicensed in northern Cyprus. Empirical treatment with cephalosporins enhanced by clavulanic acid is not recommended by the authors. The induction of AmpC enzymes in Enterobacteriaceae may inactivate the cephalosporin in bacteria. UTIs may get even more severe and course with bacteraemia. Herein, delay in adequate therapy will lead to adverse outcomes and potentially increased mortality and morbidity (Kumar et al., 2006). Intravenous antimicrobial therapy (chosen according to the susceptibility pattern of the organism) should be administered. Unfortunately, our ESBL-producing *E. coli* isolates were not tested in term of resistance against fosfomycin, which represents a current favorite choice among practitioners due to limited side effects and simplicity for the treatment of MDR *E. coli* causing UTIs in literature (Estebanez et al., 2009; Cho et al., 2015). The immediate inclusion of fosfomycin antimicrobial resistance test is needed in Cyprus.

Besides strategies for smarter antimicrobial use, several new treatment choices can be potentially administered in Cyprus, such as phage therapy, antimicrobial peptide (AMPs) therapy and immunotherapy (Haq et al., 2012; Worthington and Melander, 2013). Especially, the effective use of therapeutic bacteriophages was already started over a century ago that has been later on underestimated by the discovery of the antimicrobials (Kutateladze and Adamia, 2010; Haq et al., 2012; Keen, 2012). Mainly, Eastern European and Russian medical doctors have used the phage therapy to treat insisting MDR bacterial infections (Kutateladze and Adamia, 2010; Abedon et al., 2011). Highly specific and bacteria lysing effective phages for different *E. coli* strains have been previously published (Brüssow, 2005; Maura et al., 2012; Sillankorva et al., 2012; Tsonos et al., 2012). Furthermore, sequence-specific gene fragments that can be injected into the harmful pathogens by individually designed phages have also been lately described (Lu and Koeris, 2011; Bikard et al., 2014; Qadir, 2015). This technology creates opportunities to kill the targeted pathogens in complex bacterial populations while preventing the spread of plasmid- borne resistance genes, in endemic countries, such as Cyprus.

The majority of the *E. coli* strains causing UTIs can produce biofilms, which significantly increase resistance to antimicrobials and natural immune- system, whereas phages are able to pass through the extracellular matrix, to degrade the biofilm and kill the bacteria (Doolittle et al., 1995; Lacroix-Gueu et al., 2005).

There are also accumulating reports in the literature regarding activity of AMPs that contribute to innate immune responses and destroy the harmful pathogens such as *E. coli* (Corrales-Garcia et al., 2013; Lira et al., 2013). Those small peptides enter into membrane bilayer of the microbes and form channels resulting in cell death (Hassan et al., 2012).

In case of insisting UTIs every other aspect of diagnostic investigations (i.e., CT, Magnetic Resonance Imagining, pelvic and renal ultrasound) should be carried out to find out the complicating factors rather than simple addition of antimicrobials.

The scope of this study was limited with investigation of phenotypical ESBL-producing *E. coli* prevalence in Cyprus. Although, continuous epidemiologic data collection supported by molecular typing are needed on the entire island in future. According to recent metagenomic studies, the environment is the largest gene pool which is closely related to those conferring resistance in human pathogens (Cantas et al., 2013; Cantas and Suer, 2014). It is therefore not possible to eradicate any multi-resistant genes posing bugs on the earth. Yet routine antimicrobial resistance screenings from gut microbes isolated from environment and animals can at least contribute to a better understanding and control of possible spread of ‘super bugs’ and resistance genetic elements on the island. Each year, over two million tourists visit Cyprus. Hypothetically, new resistance genes may travel in and out of the country with potential pandemics.

## Conclusion

This study withholds the first nationwide antimicrobial resistance test records of previously isolated microbes from urine samples in Cyprus. The high frequency of ESBL-producing *E. coli* causing cystitis is an emerging problem in hospitalized and out-patients on the island. Non-resistance to amikacin, meropenem, imipenem and relatively high susceptibility to nitrofurantoin can be considered as good choices for the empirical treatment of complicated UTIs in Cyprus.

Continuous surveillance of bacterial resistance is needed to generate essential epidemiological data which promotes and directs country specific stewardship activities. Diagnostic tests should be more commonly used in routine clinical practice for targeted therapy. Ideally, patients should have access to precise information on the infectious disease, antimicrobial resistance and its consequences instead of simple and easy access to drugs without prescriptions. An immediate ban of over-the-counter sale of antimicrobials has to be implemented at each segment of healthcare. As a result, it can be argued that further work and new strategies for continuous dissemination of multidisciplinary research findings related to antimicrobial resistance development on the island is needed.

### Ethical Statements

This study was conducted in the absence of a governmental review board to approve it, but patient treatment and procedures to ensure sample anonymity followed best practices. Previous Lab records were gained by the permission of the Lab manager.

## Author Contributions

LC conceived the idea for the study, contributed to the organization and management of the project and acquisition of funds, performed the data collection, interpreted the results, and formulated the underlying causes and drafted the manuscript. KS supervised all bacteriological laboratory and drafted the manuscript. EG performed the sampling and drafted the manuscript. TI supervised the whole project and drafted the manuscript. All four authors discussed the results, revised and adopted the manuscript.

## Conflict of Interest Statement

The authors declare that the research was conducted in the absence of any commercial or financial relationships that could be construed as a potential conflict of interest.
